# DNA demethylation-dependent enhancement of toll-like receptor-2 gene expression in cystic fibrosis epithelial cells involves SP1-activated transcription

**DOI:** 10.1186/1471-2199-9-39

**Published:** 2008-04-21

**Authors:** Takashi Furuta, Tsuyoshi Shuto, Shogo Shimasaki, Yuko Ohira, Mary Ann Suico, Dieter C Gruenert, Hirofumi Kai

**Affiliations:** 1 Department of Molecular Medicine, Graduate School of Pharmaceutical Science, Global COE "Cell Fate Regulation Research and Education Unit", Kumamoto University, Kumamoto 862-0973, Japan; 2 California Pacific Medical Center Research Institute, 475 Brannan St, Suite 220, San Francisco, CA 94115, USA; 3 Department of Laboratory Medicine, University of California, San Francisco, San Francisco, CA 94143, USA; 4 Department of Medicine, University of Vermont, Burlington, VT 05405, USA

## Abstract

**Background:**

The clinical course of cystic fibrosis (CF) is characterized by recurrent pulmonary infections and chronic inflammation. We have recently shown that decreased methylation of the toll-like receptor-2 (TLR2) promoter leads to an apparent CF-related up-regulation of TLR2. This up-regulation could be responsible, in part, for the CF-associated enhanced proinflammatory responses to various bacterial products in epithelial cells. However, the molecular mechanisms underlying DNA hypomethylation-dependent enhancement of TLR2 expression in CF cells remain unknown.

**Results:**

The present study indicates that there is a specific CpG region (CpG#18-20), adjacent to the SP1 binding site that is significantly hypomethylated in several CF epithelial cell lines. These CpGs encompass a minimal promoter region required for basal TLR2 expression, and suggests that CpG#18-20 methylation regulates TLR2 expression in epithelial cells. Furthermore, reporter gene analysis indicated that the SP1 binding site is involved in the methylation-dependent regulation of the TLR2 promoter. Inhibition of SP1 with mithramycin A decreased TLR2 expression in both CF and 5-azacytidine-treated non-CF epithelial cells. Moreover, even though SP1 binding was not affected by CpG methylation, SP1-dependent transcription was abolished by CpG methylation.

**Conclusion:**

This report implicates SP1 as a critical component of DNA demethylation-dependent up-regulation of TLR2 expression in CF epithelial cells.

## Background

The innate immune system recognizes conserved microbial products, termed pathogen-associated molecular patterns (PAMPs), that are invariant among diverse groups of microorganisms. PAMPs are recognized by a set of germ-line encoded pattern recognition receptors (PRRs) including toll-like receptors (TLRs) [1-3]. After the recognition of microbial PAMPs by innate immune systems, TLRs activate signaling pathways that induce inflammatory cytokines and antimicrobial peptides to eliminate invading pathogens. Eleven members of the mammalian TLR family have been identified and cloned thus far. TLR2 and TLR4 have been studied in the greatest depth [4,5], and TLR4 appears to be primarily involved in the recognition of lipopolysaccharide (LPS) from Gram-negative bacteria [5]. In contrast, TLR2 responds to a variety of both Gram-negative and Gram-positive bacterial products, including peptidoglycan, lipoprotein, lipoteichoic acid, and lipoarabinomannan. This suggests that TLR2 plays a critical role in the host defence system [4].

TLR2 is primarily expressed in monocytes, macrophages, dendritic cells, and granulocytes. There is also a growing body of evidence indicating that TLR2 is inducibly expressed in epithelial tissues. Previous studies have shown that TLR2 is expressed at a low level in human epithelial cells under physiological conditions. However, TLR2 expression is greatly increased during bacterial infections through an NF-κB-dependent mechanism [6,7]. Inducible expression of human TLR2 by the proinflammatory cytokine, TNFα, was also indicated in lung Type II-like A549 epithelial cells [8]. These findings indicate that increased TLR2 expression during bacterial infection may contribute in accelerating the immune response to invading pathogens.

Although optimal TLR2 signaling is required to activate epithelial cells against microorganisms, excessive or inappropriate TLR2 expression and signaling could contribute to hyperresponsiveness against bacterial ligands, and enhance the inflammatory responses frequently detrimental to the host [6,9-12]. Our previous studies indicate that expression of TLR2 is higher in the airway mucosae of chronic otitis media patients than in normal subjects [6], and is also associated with inflammatory bowel disease (IBD) such as Crohn's disease [11,12]. These reports implicate hyperresponsiveness to bacterial infection as a cause of the enhanced susceptibility to chronic inflammation. Thus, regulation of TLR2 expression is likely an important immune-regulatory mechanism, commonly involved in host defence against bacterial infections.

The clinical course of cystic fibrosis (CF), the most common lethal inherited disorder in Caucasians, is characterized by two major respiratory symptoms, recurrent pulmonary infection and chronic inflammation that ultimately lead to death from respiratory failure [13,14]. Two previous studies [15,16] showed a modest up-regulation of TLR2 in CF airway epithelial cells that is consistent with an increased proinflammatory response to TLR2-activating bacterial ligands found in CF airways. In accordance with these findings, an increase in the expression of TLR2 gene in CF epithelial cells due to the hypomethylation of the TLR2 promoter has been demonstrated [17]. However, the molecular mechanisms underlying this hypomethylation-dependent activation of TLR2 transcription in human CF epithelial cells remain unknown.

The present study shows that a specific CpG region, adjacent to an SP1 binding site, are hypomethylated in CF epithelial cells. The minimal region required for maintenance of basal TLR2 promoter activity was comprised of the CpG site adjacent to an SP1 binding site. Furthermore, SP1-dependent transcriptional activity, but not SP1 binding, was shown to be elevated in the hypomethylation-dependent enhancement of TLR2 gene expression in CF epithelial cells.

## Results

### Identification of CF-specific methylation patterns within the human TLR2 promoter

**Figure 1:**
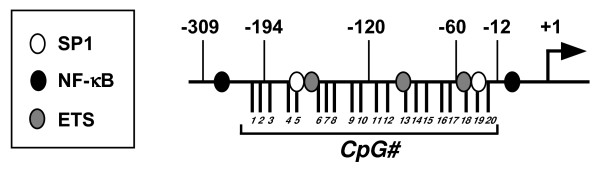
**Schematic representation of the human TLR2 promoter**. Binding sites for different transcription factors are indicated. Black lines are CpG sites that can be methylated. Transcriptional start site is indicated as +1.

We recently showed that decreased methylation of human TLR2 (hTLR2) promoter is responsible for enhanced TLR2 expression in CF epithelial cells, and suggested that this increased TLR2 expression is responsible for augmenting the proinflammatory response to various bacterial products [17]. To identify specific promoter regions among the 20 potentially hypomethylated CpG sites in CF epithelial cells, the methylation profiles of these sites were characterized by bisulfite sequence analysis. The 20 CpG sites (Fig. 1) were first bundled together into eight groups based on the relative proximity of each CpG (Table 2). The methylation status of these eight CpG groupings was then analyzed in non-CF (16HBE14o-, HeLa, A549) and CF (CFBE41o-, CFTE29o-, CFPAC-1) epithelial cells. The proportion of methylated to unmethylated sites was assessed. Statistical analysis using Fischer's exact test compared non-CF/CF cells in the context of methylated/unmethylated CpGs in each site (Table 2). This analysis identified CpG groupings #1–3 and #18–20 as significantly hypomethylated (*p *< 0.001) and CpG groupings #6–8, #13–15 and #16–17 as moderately hypomethylated (*p *< 0.05). Statistical analysis showed that the methylation of CpG groupings #4–5, #9–10 and #11–12 were not significantly different (p > 0.05) between non-CF and CF epithelial cells. These findings suggest that there are CF-specific methylation patterns (CFSMPs) within the TLR2 promoter that may play a role in CF pathology.

**Table 1:** Oligonucleotides used for this study

Primers used for the generation of TLR2 promoter/reporter constructs		
pGL3-T2P(-309)	5'-GCCTCTAGCGTCTCGATTCGC-3'	5'-CTGGGAGAACTCCGAGCAGTC-3'
pGL3-T2P(-194)	5'-CTGTCGCAGCCTAGCTCACGG-3'	5'-CTGGGAGAACTCCGAGCAGTC-3'
pGL3-T2P(-120)	5'-CCTGCCTGGAACTCAGCGCG-3'	5'-CTGGGAGAACTCCGAGCAGTC-3'
pGL3-T2P(-60)	5'-TGCCCCGTGGAAGGGGCGGTT-3'	5'-CTGGGAGAACTCCGAGCAGTC-3'
pGL3-T2P(-12)	5'-CGCGCGGACTTTCCCTTTTGC-3'	5'-CTGGGAGAACTCCGAGCAGTC-3'

Primers used for the generation of mutant TLR2 promoter/reporter constructs^*a*^		

pGL3-T2P(-120) SPm	5'-CCCGTGGAAGG*TTA*GGTTCCCGCACCCCAG-3'	5'-CTGGGGTGCGGGAACC*TAA*CCTTCCACGGG-3'
pGL3-T2P(-120)/(-60) ETSm	5'-GCGCACGTGCCCCG*AC*GAAGGGGCGGTTCC-3'	5'-GGAACCGCCCCTTC*GT*CGGGGCACGTGCGC-3'

Primers used for semi-quantitative RT-PCR		

SP1	5'-TGTGAATGCTGCTCAACTCTCC-3'	5'- CATGTATTCCATCACCACCAG -3'
SP3	5'-AACCTGATCCTGAAGAGTGGC-3'	5'-TGGCGGAAGTATTAACAGTTCC-3'

Oligonucleotides used for EMSA^*b*^		

T2P(-64/-31)	5'-GTGCCCCGTGGAAGGGGCGGTTCCCGCACCCCAG-3'	5'-CTGGGGTGCGGGAACCGCCCCTTCCACGGGGCAC-3'
T2P(-64/-31) SPm	5'-GTGCCCCGTGGAAGG*TTA*GGTTCCCGCACCCCAG-3'	5'-CTGGGGTGCGGGAACC*TAA*CCTTCCACGGGGCAC-3'
T2P(-64/-31)-monoMe	5'-GTGCCCCGTGGAAGGGGCGGTTCCCGCACCCCAG-3'	5'-CTGGGGTGCGGGAAC/M-C/GCCCCTTCCACGGGGCAC-3'
T2P(-64/-31)-triMe	5'-GTGCCCCGTGGAAGGGGCGGTTCCCGCACCCCAG-3'	5'-CTGGGGTG/M-C/GGGAAC/M-C/GCCCCTTCCA/M-C/GGGGCAC-3'

*a *Mutations introduced in the oligonucleotides are shown in italic.

*b *Putative SP1 binding sequences are underlined. Mutations introduced in the oligonucleotides are shown in italic. M-C represents methylated cytosine.

**Table 2:** Methylation of CpG sites in the TLR2 promoter is significantly different between non-CF and CF epithelial cells^*a*^

CpG#		Sequences	Methylated	Unmethylated	*p *values
#1–3	non-CF	43	46	83	
	CF	41	17	106	7.277 × 10^-5^***
#4–5	non-CF	43	18	68	
	CF	41	22	60	0.4698
#6–8	non-CF	43	8	121	
	CF	41	1	122	0.0362*
#9–10	non-CF	43	3	83	
	CF	41	1	81	0.6209
#11–12	non-CF	43	6	80	
	CF	41	1	81	0.1179
#13–15	non-CF	43	20	109	
	CF	41	8	115	0.0273*
#16–17	non-CF	43	12	74	
	CF	41	3	79	0.0282*
#18–20	non-CF	43	22	107	
	CF	41	4	119	0.0003***

^*a*^CpG methylation was scored at specific sites (CpG#1-3, 4–5, 6–8, 9–10, 1–12, 13–15, 16–17 and 18–20) in the TLR2 promoter sequence from non-CF (16HBE14o-, n = 23; HeLa, n = 10; A549, n = 10) and CF (CFBE41o-, n = 21; CFTE41o-, n = 10; CFPAC-1, n = 10) epithelial cells. The number of methylated and unmethylated sites was scored and a Fischer's exact test was used to assign *p *values (*, *p *< 0.05; ***, *p *< 0.001).

### Identification of the minimal promoter region required for basal TLR2 promoter activity in human epithelial cells

**Figure 2:**
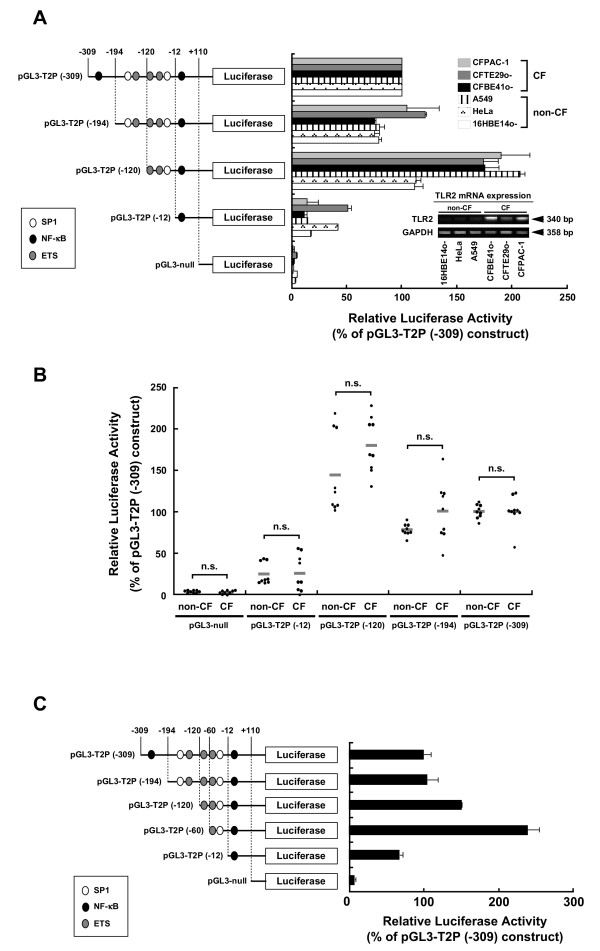
**Minimal region necessary for basal TLR2 promoter activity overlaps with the CpG sites adjacent to SP1 (CpG#18-20)**. (A) Deletion analysis of human TLR2 5'-regulatory CpG-rich region. 5'-serial deletion reporter constructs were transiently transfected into non-CF and CF epithelial cells. Luciferase activity is expressed as percent of the activity of pGL3-T2P (-309). Bar graphs represent the mean ± S.E.M. from 3 independent experiments performed in triplicate. TLR2 mRNA expression levels of each cell line are shown. (B) Comparison of non-CF and CF epithelial cell promoter activity in each deletion construct. Each data point represents luciferase activity in non-CF (16HBE14o-, HeLa, A549) and in CF (CFBE41o-, CFTE29o-, CFPAC-1) epithelial cells (n = 3 for each cell line). Mean values are represented as grey bar. The difference between non-CF and CF epithelial cells in promoter activity for each reporter construct was not significant (n.s.) as analyzed by Student's *t*-test. (C). Identification of the minimal region in the TLR2 promoter (-60/+110) necessary for basal TLR2 promoter activity. A series of 5' promoter deletion constructs were transiently transfected into HeLa cell to measure luciferase activity. Luciferase activity is expressed as the percent activity of the pGL3-T2P (-309) plasmid. Results are presented as the mean ± S.E.M. from three independent experiments performed in triplicate.

Assessment of a possible correlation between CFSMPs within the TLR2 promoter and a minimal TLR2 promoter region required for basal TLR2 promoter activity was performed by deletion analysis of the TLR2 promoter. A series of reporter plasmids containing various lengths of the promoter region were constructed (Fig. 2A). These constructs were transfected into non-CF (16HBE14o-, HeLa, A549) and CF (CFBE41o-, CFTE29o-, CFPAC-1) epithelial cells. The CF cells express high levels of TLR2, while the non-CF cells express low levels of TLR2 mRNA (Fig. 2A, RT-PCR). Luciferase activity in all cell lines decreased as a function of promoter length (i.e., transfection with plasmids pGL3-T2P (-120) through pGL3-T2P (-12)) (Fig 2A), indicating that there are primary promoter regulatory elements between -120- to -12-bp upstream of the TLR2 gene coding sequences. We noted that the pattern of luciferase activity for the different constructs in the non-CF cells seemed to be similar to the pattern of activity measured in the CF epithelial cells even though the non-CF epithelial cells show very low levels of endogenous TLR2 mRNA expression (Fig. 2A). Statistical analysis showed that the differences between non-CF and CF epithelial cells in promoter activity of the reporter constructs were not significant (Student's *t*-test, p > 0.05) (Fig. 2B). This was not an unexpected result because the TLR2 promoter sequences in CF and non-CF cells are identical.

To further restrict a minimal region required for TLR2 basal promoter activity, we constructed pGL3-T2P (-60) plasmid. This construct contains only one E26 transforming-specific (ETS) site and one SP1 binding site (Fig. 2C). The luciferase activity in the cells transfected with pGL3-T2P (-60) was 3-fold higher than in those transfected with pGL3-T2P (-12), indicating that the minimal promoter is located between -60-bp to -12-bp upstream of the transcription start site (Fig. 2C). It is important to note that this minimal region comprises CpG#18-20, and is significantly hypomethylated in CF epithelial cells. These findings support the notion that this region regulates TLR2 expression.

### SP1 binding site adjacent to CpG#18-20 in human epithelial cells

**Figure 3:**
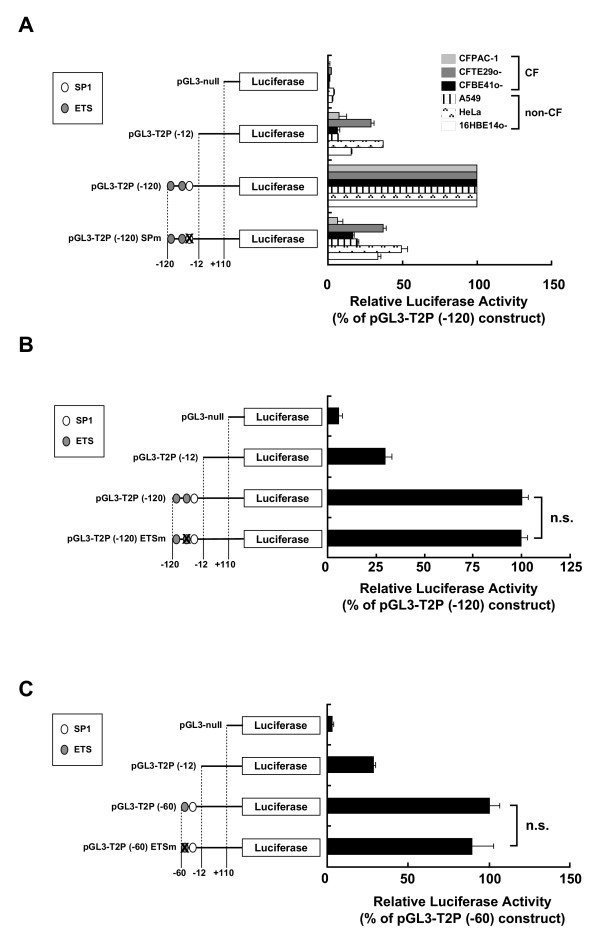
**Assessment of SP1 binding site interactions with the adjacent CpG#18-20 site**. (A) Mutation of the SP1 binding site within TLR2 promoter (-120/+110) decreases basal TLR2 promoter activity in both non-CF and CF epithelial cells. The pGL3-T2P (-120), SP1 binding site-mutant pGL3-T2P (-120) SPm, pGL3-T2P (-12), or pGL3-null plasmids were each transiently transfected into HeLa cells. Luciferase activity is expressed as the percent activity of the pGL3-T2P (-120) construct. (B) Assessment of the effect of mutating the ETS binding site within the TLR2 -120/+110 promoter region. The pGL3-T2P (-120), ETS binding site-mutant pGL3-T2P (-120) ETSm, pGL3-T2P (-12), or pGL3-null plasmids were each transiently transfected into HeLa cells. Luciferase activity is expressed as the percent activity of the pGL3-T2P (-120) construct. (C) Assessment of the effect of mutating the ETS binding site within TLR2 -60/+110 promoter region. The pGL3-T2P (-60), mutant ETS binding site- pGL3-T2P (-60) ETSm construct, the pGL3-T2P (-12) construct, or the pGL3-null plasmid were transiently transfected into HeLa cells. Luciferase activity is expressed as the percent activity of the pGL3-T2P (-60) construct. Data are presented as the mean ± S.E.M. from three independent experiments performed in triplicate. n.s. = not significant as assessed by Student's *t *test.

Since CpG#18-20 is located adjacent to putative SP1 and ETS binding sites (Fig. 1), binding to these sites was assessed to determine whether they were required for minimal promoter activity. Mutations were introduced into the SP1 and ETS binding sites in the reporter plasmids. The introduction of a SP1 mutation into the pGL3-T2P (-120) plasmid reduced the basal TLR2 promoter activity in both non-CF and CF epithelial cells (Fig 3A), indicating that this SP1 binding site is involved in modulating TLR2 basal promoter activity. On the other hand, a mutation in the ETS binding site in either the pGL3-T2P (-120) or in the pGL3-T2P (-60) construct did not affect TLR2 promoter activity (Fig. 3B and 3C).

### Regulation of TLR2 promoter activity and its gene expression by SP1

**Figure 4:**
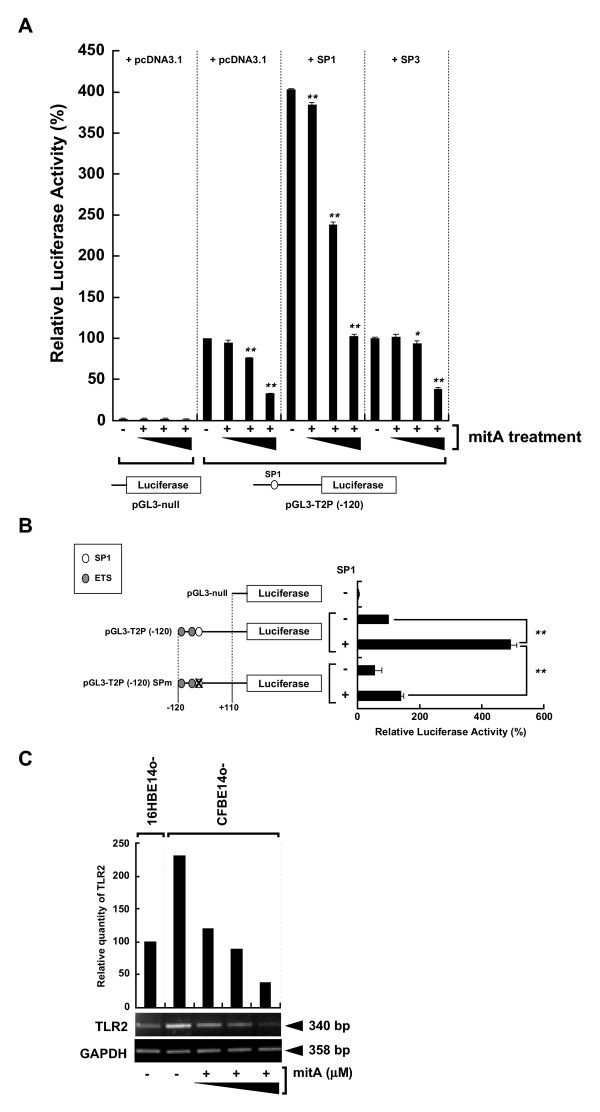
**Sp1-dependent regulation of hTLR2 promoter activity and TLR2 gene expression**. (A) Assessment of the effect of mithramycin A treatment on SP1-induced TLR2 promoter activation after co-transfecting HeLa cells with the pGL3-T2P (-120) or pGL3-null and with pcDNA3.1 empty vector the SP1 or SP3 plasmids into HeLa cells. Cells were either untreated or treated 1 hr after transfection with 50 nM mithramycin A for 24 hr. Luciferase activity is expressed as the percent activity of the pGL3-T2P (-120) plasmid in pcDNA3.1-transfected, mitA-untreated cells. (B) Assessment of the effect of mutating the SP1 binding site within TLR2 -120/+110 promoter region. The pGL3-T2P (-120) or the pGL3-T2P (-120) SPm construct was co-transfected into HeLa cells with pcDNA3.1 or SP1 plasmid. The HeLa cells were also co-transfected with the pGL3-null and pcDNA3.1 plasmids as negative control. Luciferase activity is expressed as the percent activity of the pGL3-T2P (-120) construct in pcDNA3.1-co-transfected cells. Data are presented as the mean ± S.E.M. from 3 independent experiments performed in triplicate. *, p < 0.05; **, p < 0.0001 as assessed by ANOVA with Tukey-Kramer method. (C) SP1-dependent TLR2 mRNA expression in human epithelial cells. CFBE41o- cells were either treated with mitA (0.5, 1, 5 μM) for 24 hr or untreated. Semi-quantitative RT-PCR analysis was performed using the total RNA extracted from either CFBE41o- or 16HBE14o- cells. TLR2 expression was quantified by normalizing to GAPDH (control) and assayed using Image Gauge (top of the gel images). Results are representative of three independent experiments.

SP1 binding adjacent to CpG#18-20 appears to be critical for basal TLR2 transcription, so to test the extent to which SP1 regulates TLR2 transcription, cells were treated with mithramycin A (mitA), a known Sp1-binding inhibitor [18]. Treatment of HeLa cells with mitA reduced basal TLR2 promoter activity in a dose-dependent manner (Fig. 4A, pGL3-T2P (-120) + pcDNA3.1). Furthermore, transfection of the cells with an SP1 expression vector caused a four-fold increase in basal TLR2 promoter activity that could be suppressed by mitA (Fig. 4A, pGL3-T2P (-120) + SP1). HeLa cells transfected with a plasmid, pGL3P (-120) SPm that contained a mutant SP1 binding site showed a significant reduction in SP1-induced promoter activity (Fig. 4B, *p *< 0.0001). MitA treatment of CFBE41o- cells suppressed the intrinsic TLR2 mRNA expression in a dose-dependent manner (Fig. 4C). SP3 had no effect on the basal TLR2 promoter activity (Fig. 4A, pGL3-T2P (-120) + SP3). These results indicate that SP1, but not SP3, up-regulates TLR2 promoter activity and gene expression.

### Inhibition of basal and SP1-induced TLR2 promoter activity by *in vitro *DNA methylation

**Figure 5:**
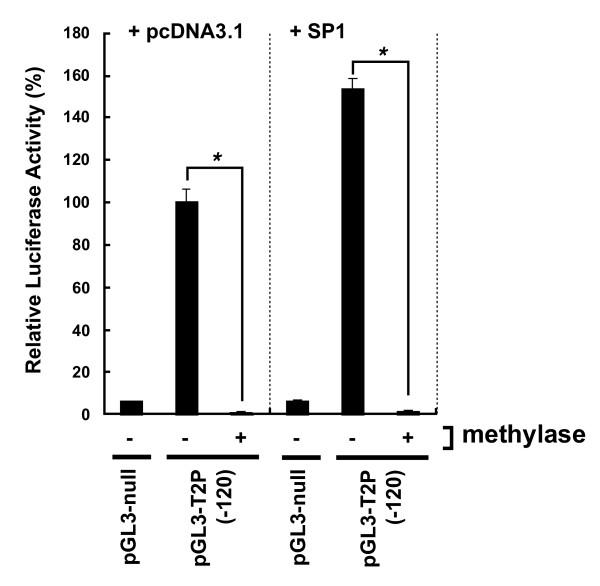
**Inhibition of hTLR2 promoter activity by DNA methylation**. *Sss*I methylase-treated or untreated pGL3-T2P (-120) was co-transfected into HeLa cells with the pcDNA3.1 or the SP1 plasmid. HeLa cells were also co-transfected with pGL3-null and pcDNA3.1 as control. Luciferase activity is expressed as the percent activity of the pGL3-T2P (-120) plasmid in pcDNA3.1-co-transfected, methylase-untreated cells. Data are presented as the mean ± S.E.M. from three independent experiments performed in triplicate. *p < 0.0001, assessed by Student's *t*-test.

The effect of *in vitro *DNA methylation on the promoter activity of human TLR2 gene was investigated through transient transfection of HeLa cells with methylated CpG reporter constructs. Endogenously activated luciferase activity was significantly reduced in the cells transfected with the methylated pGL3-T2P (-120) construct and an empty expression vector (pGL3-T2P (-120) + pcDNA3.1) (Fig. 5). Moreover, when cells were transfected with the methylated pGL3-T2P (-120) and a vector expressing SP1, TLR2 promoter activity was significantly inhibited (Fig. 5, pGL3-T2P (-120) + SP1), indicating that SP1-mediated transcriptional activation was inhibited by promoter CpG methylation.

### SP1 and TLR2 expression

**Figure 6:**
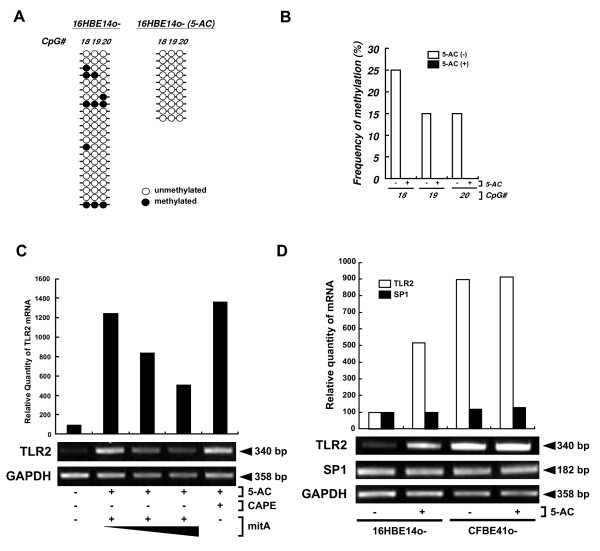
**Assessment of the effect of SP1 hypomethylation on TLR2 expression**. (A, B) 5-AC decreases CpG methylation adjacent to the SP1 binding site within human TLR2 promoter. Genomic DNA from 16HBE14o- cells was either treated with 5-AC for 7 days or untreated (n = 20 for 5-AC-untreated; n = 10 for 5-AC-treated) and then analyzed by sodium bisulfite sequencing. (A) Methylation profiles of the CpG sites (4, 5, 18, 19, 20) adjacent to the SP1 binding sites within TLR2 promoter. (B) The percent of methylated CpGs in each CpG site is shown. (C) Assessment of the effect of Mit A treatment in 5-AC-induced TLR2 up-regulation. 16HBE14o- cells were untreated or treated with 5-AC for 7 days, and then treated with mitA (0.1, 1 μM) for 24 hr or with 100 μM CAPE 3 hr. (D) SP1 expression after 5-AC treatment. 16HBE14o- and CFBE41o- cells were either treated with 5-AC for 7 days or untreated. Total RNA extracted from from both cell lines (in both (C) and (D)) and analyzed by semi-quantitative RT-PCR. TLR2 and SP1 expression was quantified by normalizing to GAPDH and assayed using Image Gauge. Results are representative of three independent experiments.

**Figure 7:**
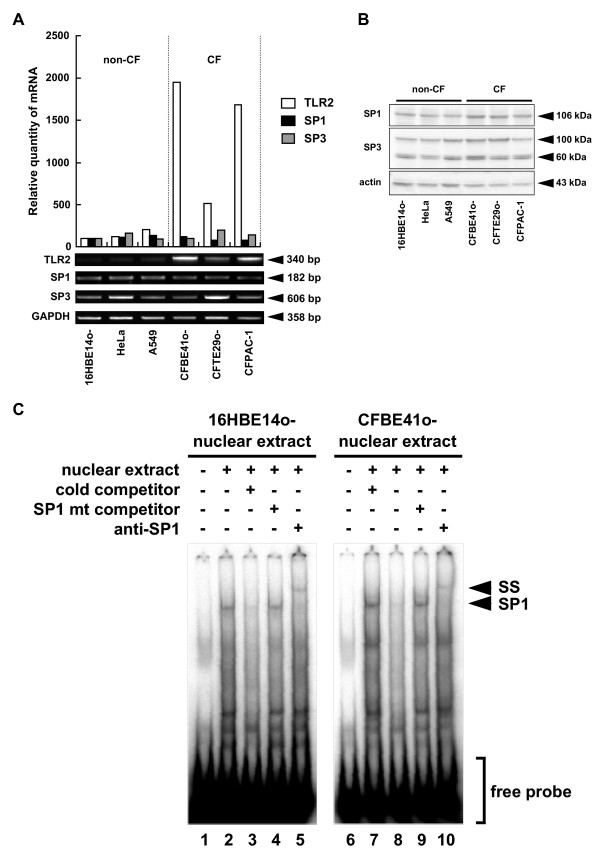
**SP1 expression in non-CF and CF epithelial cells**. (A) SP1 mRNA expression in non-CF and CF epithelial cells. The levels of TLR2, SP1 and SP3 mRNA in the indicated cell lines were determined by semi-quantitative RT-PCR analysis. Gene expression was quantified by normalizing to GAPDH and assayed using Image Gauge. Results are representative of three independent experiments. (B) SP1 and SP3 protein levels in non-CF and CF epithelial cells were determined by Western blotting of nuclear extracts. Actin was used as an internal control. Results are representative of three independent experiments. (C) Sp1 binding in 16HBE14o- and CFBE41o- cells was assayed using labeled oligonucleotides containing the CpG#18-20 site and one putative Sp1 binding site (T2P(-64/-31)). Unlabeled WT oligonucleotides (lanes 3 and 8) or unlabeled mutant SP1 oligonucleotides (Table 1, T2P(-64/-31) SPm) (SP1 mt competitor, lanes 4 and 9) were added to the reaction solution for competition analysis. Supershift analysis was performed using an SP1 antibody (anti-SP1, lanes 5 and 10) to detect SP1-specific binding. Arrows indicate SP1-containing complexes (SP1) and supershifted SP1 (SS).

16HBE14o- cells were treated with 5-azacytidine (5-AC) and mitA to assess if the endogenous methylation of DNA affects SP1-induced TLR2 expression. Treatment with 5-AC inhibited the methylation at CpG#18-20 (Fig. 6A, B) and significantly increased TLR2 expression in 16HBE14o- cells (Fig. 6C) [17]. This 5-AC-induced expression of TLR2 mRNA was inhibited by mitA (Fig. 6C). CAPE, a known inhibitor for NF-κB [19], had no effect on 5-AC-mediated expression. Although 5-AC treatment significantly increased the levels of TLR2 mRNA in 16HBE14o- cells, it did not affect endogenous SP1 expression (Fig. 6D). This indicates that 5-AC-induced up-regulation of TLR2 mRNA in 16HBE14o- cells was due to the increase of SP1-dependent transcriptional activity, but independent of an increase in SP1 expression. Consistent with this, notion is the observation that SP1 mRNA expression (Fig. 7A), the nuclear expression of SP1 protein (Fig. 7B), and SP1 binding to a TLR2 promoter probe (Fig. 7C) were all similar in these cells.

### Methylation of CpG#18-20 and SP1 binding

**Figure 8:**
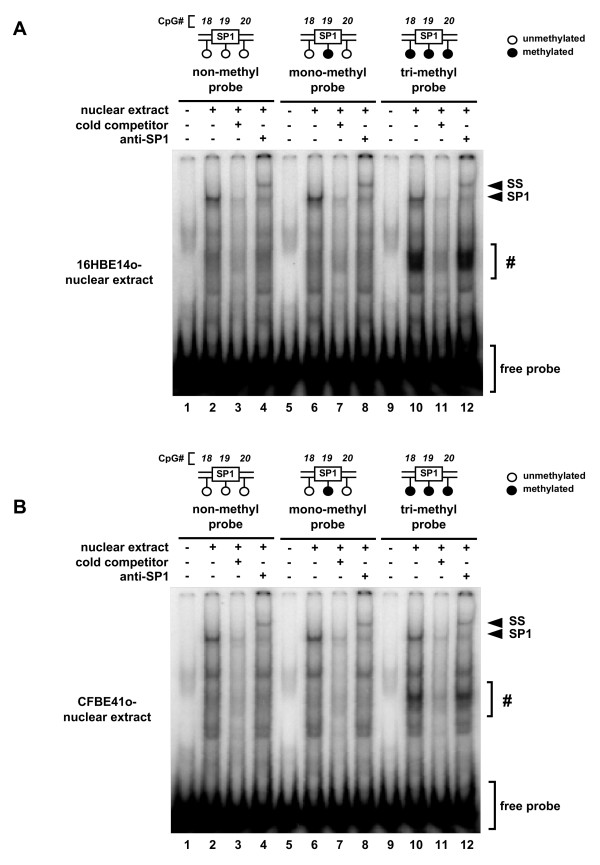
**The effect of methylation on the binding of SP1 to CpG#18-20**. Labeled oligonucleotides containing unmethylated, singly-methylated, or triple-methylated CpG sites were mixed with nuclear extract derived from 16HBE14o- (A) or CFBE41o- (B) cells and subjected to EMSA. Competition analysis was carried out by adding unlabeled oligonucleotides (lanes 3, 7 and 11) to the reaction solution. Supershift analysis to detect SP1-specific binding was performed using an SP1 antibody (lanes 4, 8 and 12). Arrows indicate the SP1-containing complexes (SP1) and the supershifted SP1 (SS). Bands derived from probes containing 3 methylated CpGs are indicated by (#).

The hypomethylation-dependent mechanisms underlying TLR2 transcription were analyzed by EMSA using unmethylated and methylated probes. Oligonucleotide probes methylated at one (mono) or three (tri) CpGs (Table 1, T2P(-64/-31)-monoMe and T2P(-64/-31)-triMe, respectively) were synthesized and subjected to EMSA analysis. SP1 from 16HBE14o- cells binds to the unmethylated, mono-methylated and tri-methylated probes (Fig. 8A, lanes 2, 6, 10). SP1-specific binding was confirmed by the supershift assay using an anti-SP1 antibody (Fig. 8A, lanes 4, 8, 12). Since the intensities of the bands from each reaction were identical, it appears that methylation at CpG#18-20 did not affect SP1 binding. Similar EMSA band patterns were also observed using nuclear extracts from CFBE41o- cells (Fig. 8B), suggesting that there is no difference in SP1 binding in non-CF and CF epithelial cells. These results indicate that mechanisms other than differential SP1 binding in non-CF and CF epithelial cells are responsible for the DNA methylation-dependent regulation of TLR2 transcription in CF epithelial cells.

## Discussion

In mammalian cells, CpG methylation in a promoter is a primary epigenetic mechanism for silencing genes, and is involved in the control of cellular function and homeostasis. It is therefore reasonable to predict that aberrant methylation of CpG regions within a promoter could manifest in a pathology associated with some chronic diseases. Recent studies have shown that TLR2 promoter hypomethylation is associated with increased expression of TLR2 in CF bronchial epithelial cells. This is consistent with CF airway pathology that typically shows increased proinflammatory responses to TLR2 bacterial ligands [17]. The present study shows that a specific CpG region of the TLR2 promoter, adjacent to an SP1 binding site, is significantly demethylated, and that this promoter region is both necessary and sufficient to maintain basal TLR2 promoter activity in human epithelial cells. These data suggest that there are CFSMPs (CF-specific methylation patterns) within TLR2 promoter, that might be used as markers to facilitate the discovery of anti-inflammatory CF drugs, as has been the case with anti-tumor drug discovery [20,21].

**Figure 9:**
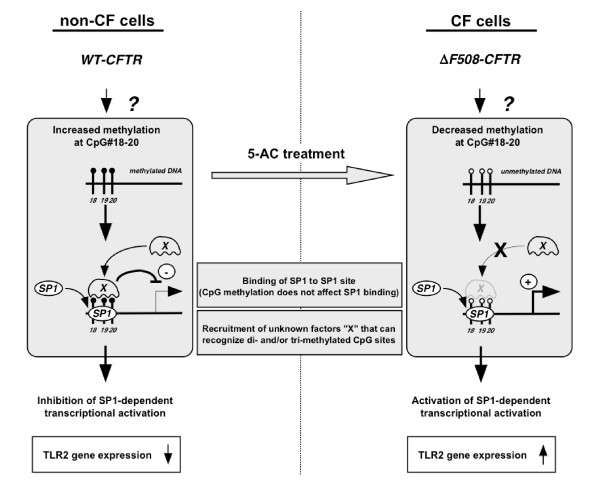
**A potential model representing the mechanisms underlying hypomethylation-dependent TLR2 promoter activation in human epithelial cells**. Enhanced methylation of the CpG#18-20 site within TLR2 promoter observed in non-CF epithelial cells is followed by the enhanced recruitment of unknown factors "X" that could recognize methylated CpGs and could suppress SP1-dependent transcriptional activation without affecting the binding of SP1 to those CpGs (left panel). 5-azacytidine (5-AC) treatment of non-CF cells leads to the demethylation of CpG#18-20. On the other hand, CF epithelial cells express high level of TLR2 gene through decreased methylation of CpG#18-20, which might abolish the recruitment and activity of factors "X" in these cells (right panel).

The studies presented here also sought to clarify the molecular mechanisms underlying demethylation-dependent enhancement of TLR2 gene expression in CF epithelial cells. SP1, but not SP3, appears to play a critical role in maintaining basal TLR2 promoter activation in human epithelial cells. Furthermore, SP1 appears to be involved in regulating DNA demethylation-dependent TLR2 transcriptional activity and expression. It is important to note that the introduction of tri-methylation into the promoter region adjacent to SP1 binding site did not abrogate SP1 binding, and suggests that SP1 binding is not rate limiting in regulating TLR2 mRNA expression. Binding of some additional unknown "X" factors to the tri-methylated probe, regardless of the source of nuclear extract, was indicated in the EMSA assay (Fig. 8A, B, lanes 10 and 12, denoted by a *sharp*). This "X" factor band was not observed in the non- or mono-methylated probes (Fig. 8A, B, lanes 2, 4, 6 and 8), suggesting that the increased methylation of CpG#18-20 in non-CF epithelial cells enhances recruitment of factors that recognize tri-methylated CpGs and suppress SP1-dependent transcription without affecting the SP1 binding (Fig. 9, left panel). On the other hand, the decreased methylation of CpG#18-20, often observed in CF epithelial cells, might abolish the recruitment and activity of these "X" factors, and allowed SP1-dependent transcription of TLR2 (Fig. 9, right panel).

The modulation of SP1 binding to its target sequences by DNA methylation is controversial. In the promoters of p21^Cip1^, 11-hydroxysteroid dehydrogenase type 2, and GSTP1 (glutathione S-transferase p1), SP1 binding was found to be diminished by DNA methylation [22-24]. In contrast, SP1 binding to the claudin-4 (CLDN4) promoter was not influenced by DNA methylation [25]. It was found that the methyl-CpG-binding domain protein 2 (MBD2) was recruited to CpG sites and silenced the CLDN4 gene in ovarian cancer cell lines without interfering with SP1 binding. The present study clearly indicates that SP1 binding to a TLR2 promoter region was not inhibited by DNA methylation. This is similar to what was observed for the CLDN4 promoter [25]. Recruitment of methyl binding proteins to the SP1 binding site in the TLR2 promoter needs to be verified. MBD2 and/or the methyl-CpG-binding protein 1 (MeCP1) complex, a large protein complex that includes MBD2 [26], could be candidate "X" factors that interact with the methylated TLR2 promoter.

It is still unclear how the methylation patterns of the TLR2 promoter are determined in non-CF and CF epithelial cell lines. Whether it is recruitment of factors required for transcriptional activation or the recruitment of factors required for silencing requires further study. The A549 and HeLa, cell lines used as non-CF epithelial cells in this study, are not thought to express CFTR mRNA, yet their TLR2 expression was comparable to that of the 16HBE14o- cells. Therefore, it appears that wt CFTR expression may not be the only factor that would determine the TLR2 gene expression in epithelial cells. However, the processing of ΔF508CFTR might affect the transcription of the TLR2 gene through a mechanism that involves the methylation of DNA and the expression of specific transcriptional regulatory factors. Preliminary studies comparing CF and non-CF cells showed no difference in the expressions of DNA methyl transferase genes, DNmt1, DNmt3a, and DNmt3b [27,28], or in the DNA demethylases, MBD2 and Gadd45a [27,29], (unpublished data). One possible mechanism could involve the recruitment of factors that releases the methylation-induced transcription block by activating demethylation of the promoter in the nucleus. Clearly, further investigation is required to elucidate these mechnaisms.

## Conclusion

There appear to be CF-associated methylation patterns within the human TLR2 (hTLR2) promoter, that lead to increased expression of the TLR2 gene in CF epithelial cells. Moreover, SP1-dependent transcription apppears to be an important component of the molecular mechanisms underlying CFSMPs-related aberrant regulation of TLR2 expression in CF epithelial cells. Increased responsiveness to TLR2 ligands as well as an increase in TLR2 expression in CF epithelial cells have been proposed as a contributing factor in CF-associated chronic inflammation. Therefore, CF-specific methylation patterns within the TLR2 promoter may have important implications for the development of therapies directed at sites regulating TLR2 expression. Furthermore, the studies presented here indicate that CF-specific hypomethylation of the hTLR2 promoter may influence regulation of other inflammation-associated genes that are aberrantly regulated in CF epithelial cells [30-34]. Further study of this system may provide insight into the molecular mechanisms regulating inflammation in CF. This study underscores the role that epigenetic mechanisms like DNA methylation play in the modulation of gene expression in response to cellular insult.

## Methods

### Reagents

5-azacytidine was purchased from Nacalai Tesque (Japan). Mithramycin A was purchased from Sigma (St. Louis, MO, USA). Caffeic acid phenethyl ester (CAPE) was purchased from Calbiochem (Darmstadt, Germany).

### Cell culture

16HBE14o- [35], CFBE41o- [36] and CFTE29o- [37] cells were previously generated and grown in Fibronectin/Vitrogen/BSA-coated flask in MEM (Invitrogen, Carlsbard, CA) [38]. CFPAC-1 cells [39] were purchased from ATCC and grown in Iscove's modified Dulbecco's medium (IMDM) (Invitrogen). The media were supplemented with 10% fetal bovine serum (FBS), 100 mg/ml of penicillin and 100 U/ml of streptomycin. The CFBE41o-, CFTE29o- and CFPAC-1 cells were derived from CF patients that are homozygous for the ΔF508 CF transmembrane conductance regulator (ΔF508CFTR) mutation. The non-CF epithelial cells in this study, 16HBE14o-, A549 and HeLa, were homozygous for wild type (wt)CFTR. Expression of CFTR mRNA in A549 and HeLa cells has not been confirmed.

### Sodium bisulfite DNA Sequencing

Genomic DNA was isolated from various cell types using the Blood and Cell Culture DNA Mini kit (Qiagen, Bothell, WA, USA) according to the manufacturer's protocol. Sodium bisulfite DNA sequencing analysis of the TLR2 promoter has been previously described [17]. Briefly, PCR products were subcloned into the pCR2.1-TOPO vector (Invitrogen, Carlsbad, CA, USA), and > 10 clones from each cell line were sequenced. The frequency of methylated CpG at each CpG site and the average % methylation at each CpG site in the TLR2 proximal promoter were calculated. The amount of methylated and unmethylated DNA at each CpG site was compared in non-CF and CF epithelial cells, and then statistically analyzed using Fischer's exact test.

### Plasmids

Full-length human TLR2 (hTLR2) promoter constructs, were generated by amplifying different lengths up to 309-bp 5' of the TLR2 gene coding region using genomic DNA prepared from A549 cells as the template for High Fidelity AccuPrime™ Taq DNA Polymerase (Invitrogen). The PCR primers used in the amplification are indicated in Table 1. The PCR products generated were cloned into pCR2.1-TOPO vector using the TOPO-TA cloning kit (Invitrogen). The TLR2 promoter-luc reporter construct was generated by subcloning the TLR2 promoter fragment into the *Xho*I-*Hind*III sites of the pGL3-Basic vector (Promega, Madison, WI, USA). Mutant TLR2 promoter constructs were generated utilizing the QuikChange II site-directed mutagenesis kit from Stratagene (La Jolla, CA, USA) according to the manufacturer's instructions. The oligonucleotide primers used to generate the mutants are shown in Table 1. Mutant constructs were generated using either the pGL3-T2P(-120) or the pGL3-T2P(-60) construct as template. The pGL3-T2P(-120) plasmid was either methylated or mock-methylated in the presence or absence, respectively, of *Sss*I methylase. Construct methylation was confirmed by digestion with methylation-dependent *Hap*II and methylation-independent *Msp*I enzymes. The TLR2 expression plasmid and the luciferase reporter construct, IL-8-luc, were described previously [40]. The NF-κB-luc construct was purchased from Stratagene (La Jolla, CA, USA). Expression plasmids for SP1 and SP3 were kindly provided by Dr. G. Suske [41]. Plasmid constructs used in this study were sequenced with ABI3730XL DNA sequencer at the genomics facility of Macrogen (Seoul, South Korea).

### Transient transfection and luciferase assay

Transient transfections with the pGL3-T2P plasmids, NF-κB-luc and IL-8-luc reporter constructs were carried out using the TransIT-LT1 transfection reagent (Panvera, Madison, WI, USA) as previously described [19]. In brief, subconfluent cells in 12-well plates were transfected with 0.4 μg reporter plasmid and 10 ng of the *Renilla *luciferase vector (phRG-TK; Promega). The empty pcDNA3.1 vector was used as a control for the TLR2 and SP1/3 transfection experiments. After 48 hrs, the transfected and control cells were harvested and luciferase activity was measured by the Dual-Luciferase Reporter Assay system (Promega) in a luminometer. Relative luciferase activity is presented as % activity of each control described in each figure legend. Values shown are the mean ± S.E.M. (n = 3).

### Semi-quantitative RT-PCR analysis

Total RNA from human epithelial cells was isolated using ISOGEN (NIPPONGENE, Tokyo, Japan) according to the manufacturer's instruction. Semi-quantitative RT-PCR was carried out using the RNA-PCR Kit (TaKaRa, Tokyo, Japan). PCR amplifications of TLR2 and GAPDH was performed as previously described [42]. The amplification conditions for SP1 and SP3 are as follows: 94°C for 60 s, 60°C for 60 s, and 72°C for 60 s (for 24 cycles). The oligonucleotide primers used in the PCR amplifications are as indicated (Table 1). Quantitative analyses were performed by using Image Gauge (FUJI FILM, Tokyo, Japan).

### Western blotting

SP1 and SP3 protein expression was assayed as previously described [18]. Equal amounts of nuclear protein extract (30 μg) were fractionated by 8% SDS-polyacrylamide gel electrophoresis and transferred to polyvinylidene difluoride membrane (Millipore, Bedford, MA, USA). The membrane was blocked with PBS-T (PBS, 0.1% (v/v) Tween 20) and 5% nonfat milk. Detection of SP1 and SP3 with anti-SP1 and anti-SP3 rabbit polyclonal antibodies (Santa Cruz Biotechnology, Santa Cruz, CA, USA) was carried as described [18]. Actin expression was determined using a goat anti-actin polyclonal antibody (Santa Cruz Biotechnology) as the internal control.

### Electrophoresis mobility shift assay (EMSA)

EMSA was performed as described [18]. Briefly, nuclear extracts (7.5 μg) from 16HBE14o- and CFBE41o- cells were incubated with [γ-^32^P]-labeled oligonucleotides representing TLR2 promoter region -64/-31 with or without the indicated excess of unlabeled competitors. The DNA-protein complexes were resolved in 4.5% polyacrylamide gels. The SP1 antibody (4 μg) was used for supershift assays, and the migrating bands were visualized autoradiographically using BAS-2000 (Fuji film, Japan). The oligonucleotides used in the EMSA are as indicated (Table 1).

### Statistical analysis

Statistical analysis of luciferase activity was performed by one-way ANOVA with either Tukey-Kramer or Dunnett's multiple comparison test (JMP software, SAS Institute, NC, USA) as indicated in each figure legends. Statistical analysis of methylation (Tables 2 and 3) was performed by the Fischer's exact test to assign *p *values.

## Authors' contributions

TF and TS participated in the conception and the experimental design, helped to draft the manuscript and carried out the experiments. SS and YO helped in carrying out the experiments and were involved in the interpretation of the data. MAS and DCG drafted the manuscript, helped in the interpretation of the data and contributed to the intellectual content. HK first conceived of the concept, designed the work, was involved in the interpretation of the data, coordinated the work and contributed to the intellectual content.
